# Understanding the failure of a behavior change intervention to reduce risk behaviors for avian influenza transmission among backyard poultry raisers in rural Bangladesh: a focused ethnography

**DOI:** 10.1186/s12889-016-3543-6

**Published:** 2016-08-24

**Authors:** Nadia Ali Rimi, Rebeca Sultana, Kazi Ishtiak-Ahmed, Md Zahidur Rahman, Marufa Hasin, M. Saiful Islam, Eduardo Azziz-Baumgartner, Nazmun Nahar, Emily S. Gurley, Stephen P. Luby

**Affiliations:** 1 Program for Emerging Infections (PEI), Infectious Diseases Division (IDD), icddr,b, 68, Shaheed Tajuddin Ahmed Sharani, Mohakhali, Dhaka, 1212 Bangladesh; 2 Centers for Disease Control and Prevention (CDC), Atlanta, GA USA; 3 Stanford University, Stanford, CA USA

**Keywords:** Backyard poultry, Behavior change intervention, Avian influenza, Focused ethnography, Bangladesh

## Abstract

**Background:**

The spread of the highly pathogenic avian influenza (HPAI) H5N1 virus among poultry and humans has raised global concerns and has motivated government and public health organizations to initiate interventions to prevent the transmission of HPAI. In Bangladesh, H5N1 became endemic in poultry and seven human H5N1 cases have been reported since 2007, including one fatality. This study piloted messages to increase awareness about avian influenza and its prevention in two rural communities, and explored change in villagers’ awareness and behaviors attributable to the intervention.

**Methods:**

During 2009–10, a research team implemented the study in two rural villages in two districts of Bangladesh. The team used a focused ethnographic approach for data collection, including informal interviews and observations to provide detailed contextual information about community response to a newly emerging disease. They collected pre-intervention qualitative data for one month. Then another team disseminated preventive messages focused on safe slaughtering methods, through courtyard meetings and affixed posters in every household. After dissemination, the research team collected post-intervention data for one month.

**Results:**

More villagers reported hearing about ‘bird flu’ after the intervention compared to before the intervention. After the intervention, villagers commonly recalled changes in the color of combs and shanks of poultry as signs of avian influenza, and perceived zoonotic transmission of avian influenza through direct contact and through inhalation. Consequently the villagers valued covering the nose and mouth while handling sick and dead poultry as a preventive measure. Nevertheless, the team did not observe noticeable change in villagers’ behavior after the intervention. Villagers reported not following the recommended behaviors because of the perceived absence of avian influenza in their flocks, low risk of avian influenza, cost, inconvenience, personal discomfort, fear of being rebuked or ridiculed, and doubt about the necessity of the intervention.

**Conclusions:**

The villagers’ awareness about avian influenza improved after the intervention, however, the intervention did not result in any measurable improvement in preventive behaviors. Low cost approaches that promote financial benefits and minimize personal discomfort should be developed and piloted.

## Background

The spread of the highly pathogenic avian influenza (HPAI) H5N1 virus among poultry and humans has raised global concerns and has motivated government and public health organizations to initiate interventions to prevent the transmission of HPAI in different countries [1–9]. In 2006, the Government of Bangladesh adopted a national pandemic influenza preparedness plan that included risk communication through mass media, workshops, posters, and leaflets, and disseminated a set of 10-step messages to prevent poultry to human transmission nationwide [10, 11]. A nationwide survey conducted in 2007 showed that 30 % of backyard poultry raisers reported having heard of avian influenza; among those who heard, 53 % did not know any of its signs, 78 % did not know how birds contracted the virus, and the most frequently (38 %) mentioned route of human infection was an incorrect belief that people were infected by eating meat or eggs of infected poultry [12]. Backyard poultry raisers are rural residents, who raise indigenous breeds with less than 50 free-range chickens, ducks, and/or geese per flock reared around the family’s domicile [13]. A subsequent qualitative study among backyard poultry raisers conducted in 2008 found that even when the Government of Bangladesh’s preventive messages reached the community, backyard raisers either did not know about avian influenza or did not believe that avian influenza could infect humans and most continued their usual practices of handling and slaughtering of sick poultry and disposal of dead poultry [14].

A principal pathway of human infection with the HPAI virus is close contact with infected birds [15–17]. Handling and slaughtering of sick and dead poultry has been associated with many human cases of H5N1 and has been identified as some of the most risky behaviors for contagion [15, 18–22]. Bangladesh has reported 549 confirmed poultry outbreaks of HPAI H5N1 in 52 out of 64 districts from 2007 to 2013 [23] and seven human H5N1 cases from 2008 to 2015, including one fatality; all of these cases were exposed to slaughtering of infected poultry [24–28]. Slaughtering sick birds is a common practice in Bangladesh [13, 14]. Preventing rural raisers from consuming sick poultry appears difficult, since poultry are a valued resource to the raisers [29]. These low-income households recover some of their financial loss by consuming sick poultry [14], which appears to be a more salient issue for these raisers than avoiding an improbable H5N1 infection. Studies in other low-income settings have also reported practices of rejecting standard recommendations or adopting risky strategies, including slaughtering sick poultry, to limit financial losses despite mass communication campaigns on avian influenza prevention [2, 6–9, 30].

In order to make behavior change campaigns more context-appropriate and feasible for low-income communities, it is important to understand villagers’ perception about their risk and about the standard preventive recommendations. Ethnographic research can inform the development of assessment tools as well as provide fertile details to design or evaluate interventions by contextualizing beliefs and behaviors [31]. Compared to a traditional ethnographic approach, the focused ethnographic method is useful to explore perceptions or behaviors pertaining to a specific area from an emic perspective, that is the local community’s perspective, among a limited number of people within a shorter period of time [32]. The relationships between knowledge or perception and specific behaviors can be best understood from situations in which the relevant behavior is directly evident (e.g., when villagers actually slaughter sick poultry). These observations can provide detailed contextual information on community response to a newly emerging disease [33] and nuanced understanding of the strengths and limitations of interventions targeted for such a disease. This study piloted recommendations designed to increase awareness about avian influenza and preventive practices to reduce risk of transmission from poultry to humans in two rural communities, and used a focused ethnographic approach to explore change in villagers’ awareness and behaviors attributable to the intervention, and the acceptability and feasibility of the recommended actions.

## Methods

### Study sites

We conducted this study in two rural villages, one from each of the districts of Rajshahi and Chittagong from June to August 2009. Rajshahi is the largest and Chittagong the third largest poultry raising area in Bangladesh [34]. We selected these two sites to capture practices in two geographically and socio-culturally distinct places of the country. We purposively selected the villages for their small size, accessibility, and being typical of the region in terms of demographic and geographic characteristics, i.e., agriculture as the main occupation, a Muslim majority and located in floodplains. The villages had not reported any avian influenza outbreak when they were selected.

### Developing intervention recommendations and materials

**Fig. 1:**
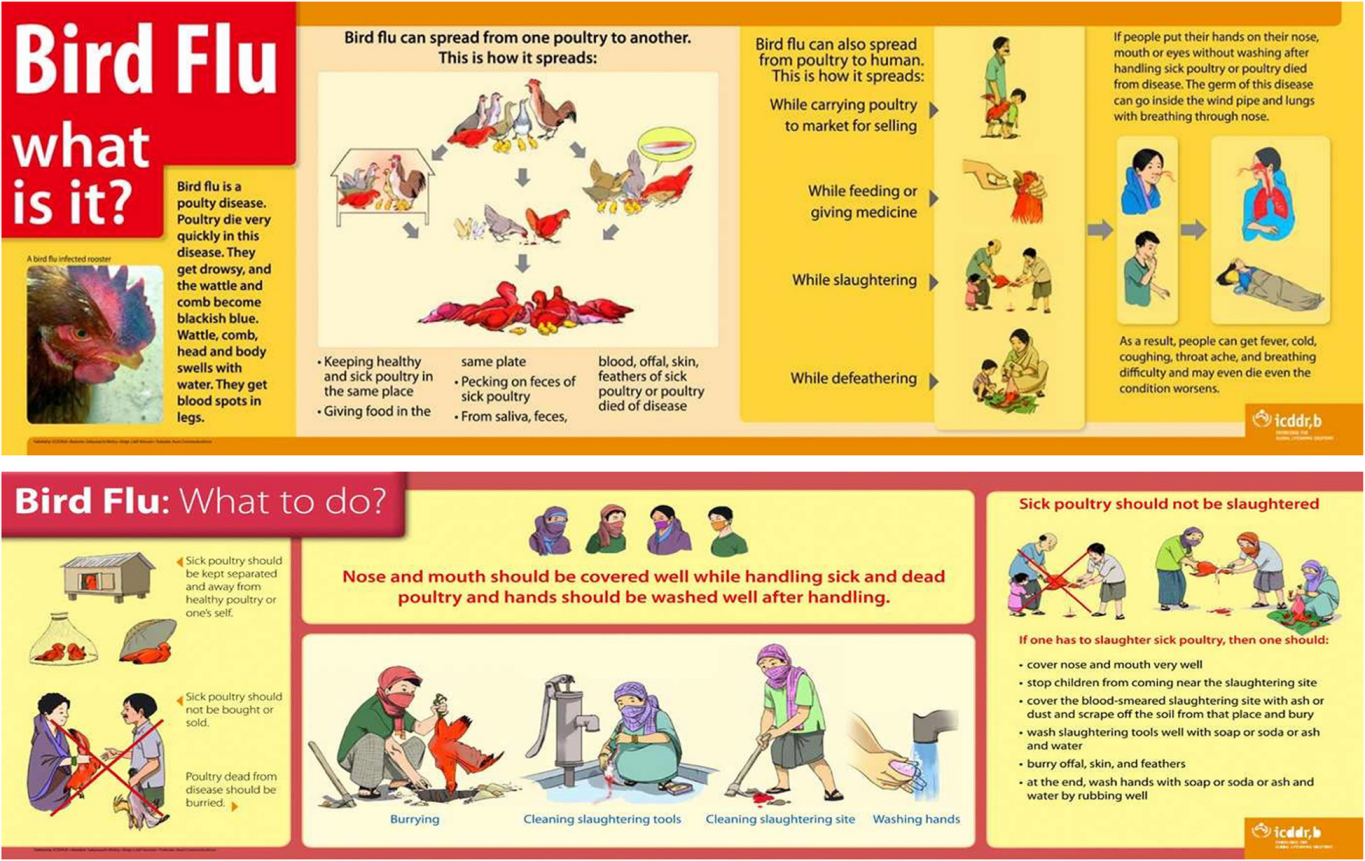
English translation of intervention posters disseminated in Rajshahi and Chittagong study villages, 2009. Reprinted from [57] under a CC BY license, with permission from icddr,b, original copyright 2009

We, a multidisciplinary team of researchers made up of anthropologists, epidemiologists, physicians, a veterinarian, a sociologist and a communication specialist, developed a set of awareness and preventive messages about avian influenza. We made the messages context-appropriate by including messages describing avian influenza disease, routes of transmission, and recommendations for handling sick and dead poultry and safer slaughtering of sick poultry, since we knew from our previous studies that villagers were not aware about avian influenza transmission and consumed sick poultry. The recommendations focused on slaughtering sick poultry, the practice believed to carry the highest risk for transmission from poultry to humans. We aimed to take a step beyond standard recommendations [10] and worked specifically on those recommendations that could be made more feasible and acceptable. For example, we took into account the financial concerns of the raisers for the recommendation ‘do not remove feathers or slaughter or handle infected birds at home’ and recommended safe slaughtering steps to minimize raiser’s financial loss. Then we produced a flipchart with illustrations in the form of a story of an outbreak of avian influenza set in a rural village and two posters (83 cm × 28 cm) with summarized messages (Fig. 1). In the flipchart and posters, we used illustrations that low-literacy rural raisers could understand. We field-tested the posters with individuals at villagers’ literacy level to confirm if they understand the messages. We included risky practices we found were relevant in previous qualitative exploration among backyard poultry raisers as routes of transmission from poultry to humans [14, 29]. We recommended locally available materials, such as a towel (*gamchha*), women’s scarf (*orna*) and typical women’s clothing in rural Bangladesh (*sharee*) to cover the nose and mouth and recommended handwashing because of unavailability and cost of masks and gloves. We used these locally available materials in the illustrations. The intervention always referred to both sick ducks and sick chickens as ‘sick poultry’. Asymptomatic birds were not part of the messaging.

### Data collection before intervention

A team of five native Bengali anthropologists and sociologists (the ‘research team’, NAR, KI, MZR and MH) lived in the study villages during the entire study period and participated in villagers’ daily life to observe and understand villagers’ awareness of avian influenza and their practices of handling, buying, selling and slaughtering of sick poultry and handling dead poultry. As part of the focused ethnographic approach, the research team collected pre-intervention data for one month using observations and informal interviews [35] until they reached data saturation on each topic (Table 1). To ensure consistency across the interviews and observations, the team discussed findings and reviewed guidelines at the end of each day. In each village, a male and a female member of the research team visited every household to collect information on demographics (number of members in the household and age, sex, religion and education of each member) and number of poultry raised by the household. They counted the total number of backyard poultry in both villages at the beginning and at the end of the first month. During these visits, the researchers also requested the villagers to inform them of any sick and dead poultry related activity in their household or neighborhood either in person or over phone. Drawings and materials used in the illustrations and language for the dissemination were revised to ensure appropriateness for the target audience.

**Table 1:** Total number of informal interviews and observations used to explore different topics with informants in Rajshahi and Chittagong villages, Bangladesh, 2009

Topics	Before intervention		After intervention	
	Informal interviews^a^	Observations^b^	Informal interviews^a^	Observations^b^
Awareness on avian influenza	42	N/A	36	N/A
Housing sick poultry	27	3	33	2
Selling/buying sick poultry	20	0	22	2
Disposing carcasses or offal/blood of sick poultry	46	6	45	7
Slaughtering sick poultry	42	6	49	8
Cleaning site/tools after slaughtering sick poultry	8	3	13	4
Hand hygiene after slaughtering/handling sick/dead poultry	9	6	16	9
Covering nose/mouth while slaughtering/handling sick/dead poultry	0	6	14	9
Keeping children away from slaughtering site	14	3	3	4

^a^Total numbers of interviews with individuals to explore each topic

^b^Total number of opportunities to observe practices pertinent to each topic

#### Observations

Throughout the fieldwork, observation was the most important tool for data collection. The researchers conducted observations whenever they encountered slaughtering of sick poultry and/or handling of sick or dead poultry or when called upon by the villagers. The researchers conducted the observations using a guideline (Appendix 1) to take notes on handling, slaughtering, disposal, hygiene practices and the presence of children during the events. No set time duration was followed for an observation session.

#### Informal interviews

The researchers conducted informal interviews with members of households, where they conducted observations and other poultry raising households. Researchers selected the informants purposively based on their role in raising or slaughtering poultry and their willingness to be interviewed. Researchers often conducted informal interviews immediately after observations. They conducted face-to-face interviews at informants’ preferred time and location, usually in their dwellings. Some interviews were scheduled, while others occurred spontaneously, for example, during villagers’ free time, or after a slaughtering or while transporting a sick poultry for selling. The team used a guideline (Appendix 2) to explore different topics. Probing questions were often derived from the participants’ responses thus the same probing questions were not always asked of all participants. The topic of an interview session often depended on a particular event relating to sick or dead poultry either observed by the researchers or informed by the villagers. The interviews were conducted as conversations with a natural flow instead of a structured question-answer session with fixed duration. Information collected through observations was often explored during interviews. The research team took field notes and recorded the informal interviews using audio recorders. The team also maintained ethnographic diaries to record daily detailed field notes throughout the fieldwork which helped to contextualize the data.

### Intervention

After one month, a separate team of three anthropologists and sociologists (the ‘intervention team’, RS and MSI), invited all villagers and disseminated the intervention messages through hour-long courtyard meetings where they used the story flipchart (two meetings in the village in Rajshahi and three in the village in Chittagong). During these meetings, the intervention team also mentioned the consequences of not following the preventive practices with an idea of number of poultry and humans infected in avian influenza all over Bangladesh. We used separate teams for intervention and data collection to reduce bias that might have resulted from being observed or interviewed by the same person who disseminated the recommendations. Children’s participation was particularly emphasized during these meetings because they play role in disseminating information among their family members. The intervention team used local terms during the dissemination. The intervention team conducted the meetings using an interactive approach where villagers asked questions and the intervention team answered the villagers’ queries. After the meeting, the intervention team provided and affixed posters (Fig. 1) on a frequently visible wall inside or outside all households in each of the study villages and left the village after dissemination.

### Data collection after intervention

After the dissemination, there was no reminder of the intervention except the presence of posters and the research team in the communities. Starting immediately after the intervention, the same research team collected data for one month using the same methods as before the intervention (Table 1). The research team looked for indications of acceptability and feasibility of the intervention recommendations during their interviews and observations. While wandering through the villages, the team had many impromptu conversations which provided them with opportunities to listen to villagers’ rationales of why they were not complying with certain recommended measures.

At the end of each day of data collection, the research team shared experiences among themselves. They discussed the queries they received and the responses given to villagers to assure they maintained uniformity in their responses by only referring to the messages disseminated by the intervention team. The research team built rapport with the villagers to gain their trust; this helped them respond to negative remarks and reactions from the villagers.

In January 2010, five months after the intervention and four months after the research team had moved out of the villages, Rajshahi villagers reported a poultry die-off of an unknown cause. The research team returned to the village and collected data through 45 informal interviews and four observations of handling of sick and dead poultry in the Rajshahi village for a week. They explored information about the poultry illness and whether there was any change in the handling and slaughtering of sick and dead poultry.

### Data analysis

The research team completed the field notes and transcribed the recorded data verbatim in Bengali. They performed thematic analysis [36] of the field notes and transcripts. First, they read the transcriptions and field notes repeatedly to get a sense of the data set. Four members (NAR, KI, MZR and MH) of the research team individually came up with lists of codes (e.g., signs of avian influenza, routes of transmission from poultry to humans, slaughtering sick poultry, disposing poultry carcass) from the data and then discussed together to develop a more comprehensive code list that included all basic segments of the raw data that could be assessed in a meaningful way. They coded all data using Atlas.ti software while modifying the code list further to allow emerging themes. The first author sorted the different codes into potential themes and sub-themes (e.g., change in awareness after intervention, awareness of avian influenza, rationale for ignoring recommended behavior), and combined all the relevant coded data within the identified themes relevant to the study objectives. Then the first and second author reviewed and refined the themes to form a coherent pattern themselves and in relation to the entire data. They looked for similarities and patterns, and took variations and context into consideration for analysis and prepared summaries for each theme. They analyzed the data by selecting vivid compelling examples from the data and relating back to the research objective and literature. They used illustrative quotes that reflected the authenticity of the collected information to highlight particular themes. They triangulated data from interviews and observations.

## Results

### Demographic information

Among the 466 residents from 114 households in the of the Rajshahi village, 73 % were Muslim and 27 % were Hindu; 27 % had more than primary education. The total population of the Chittagong village was 737 from 138 households, all were Muslim and 34 % had more than primary education. In both villages, most households raised backyard poultry [13]. Their main source of income was crop farming. In June-July 2009, the average number of backyard poultry in poultry raising households was 987 in the Rajshahi village (with an average of nine poultry per household) and 993 in the Chittagong village (with an average of eight poultry per household). Our informants were mostly women, who mainly carried out poultry-related activities and were the major caregivers and decision makers for poultry within that household. Some of our informants were men, who the research team observed slaughtering poultry. Sixty two percent of informants attended school; 37 % were between grade 1 and 5, and 25 % attended grade 6 and above.

### Change in awareness

More villagers reported hearing about ‘bird flu’ after the intervention compared to before the intervention (97 versus 29 %) (Table 2). Before the intervention, informants mentioned bird flu as a disease of broiler or commercial farm chickens or a foreign disease, which does not occur in Bangladesh. A few informants recalled one or two signs and reported that sudden death was a sign that a flock was infected with bird flu. After the intervention, villagers mentioned that bird flu could infect all kinds of poultry, including their home-raised chickens. They rarely mentioned ducks while recalling messages about avian influenza. Informants recalled nine signs but most recalled change in the color of combs and shanks on poultry, for example, comb turning blue or black and the shank turning blood spotted or black. After the intervention, more villagers reported that bird flu could be transmitted from poultry to poultry compared to before the intervention (53 versus 6 %) and mentioned routes of transmission that were relevant to their everyday practices, such as keeping healthy poultry in the same place with sick poultry or giving food to healthy poultry from the same pot as sick poultry. Reporting on the possibility of transmission of bird flu from poultry to humans also increased after the intervention compared to before the intervention (78 versus 19 %). Informants most frequently (18/25) mentioned inhaling contaminated ‘*gas*’, ‘*gondho*’ (smell), or ‘*batash*’ (air) as a route of transmission to humans and associated this route with the preventive recommendation of covering the nose and mouth. More informants reported preventive measures after the intervention compared to before the intervention (81 versus 19 %) (Table 3). Covering the nose and mouth while slaughtering sick poultry, burying carcasses and offal, were the two most frequently mentioned preventive measures after the intervention. Television was the predominant reported source of information about avian influenza before our intervention; however 63 % (22/35) of people reported the intervention as the only source of information about avian influenza. Villagers spoke spontaneously about the posters and could recall the messages from the posters. Those, who were literate, could read and explain the posters. Those, who could not read, could explain the illustrations.

**Table 2:** Awareness of avian influenza disease and its route of transmission before and after intervention, Rajshahi and Chittagong villages, Bangladesh, 2009

Topics *[Intervention messages]*	Interviews before intervention (*N* ^a^ = 42)			Interviews after intervention (*N* ^*^ = 36)		
	Responses	Number of interviews with responses	(%)	Responses	Number of interviews with responses	(%)
Heard/knew about bird flu disease *[Bird flu is a poultry disease]*	- A disease of broiler/farm poultry, not of backyard poultry - Many poultry died/killed by government - Conspiracy of foreign country - A disease of chickens, not ducks - A birds’ disease caused from flu/cold - A foreign disease/did not occur in our village/country	12^b^	(29)	- A disease of poultry/chicken/backyard chicken, which can also infect humans - A hazardous infectious virus of chickens which can also infect humans	35^b^	(97)
Signs in poultry *[Poultry die very quickly in this disease. They get drowsy, and the wattle and comb become blackish blue. Wattle, comb, head and body swells with water. They get blood spots in legs.]*	- Sudden death - Fever - Gizzard melts and chickens die in 24 h	3^b^	(7)	- Blue/blackish wattle/comb - Blood/black spots in leg - Drowsiness/sit quiet - Swollen body - Fever - Liquid defecation - Stop eating - Secret saliva - Cold/coughing	24^b^	(67)
Can transmit from poultry to poultry *[Bird flu can spread from one poultry to another]*		6	(14)		19	(53)
Route of transmission from poultry to poultry *[Keeping healthy and sick poultry in the same place; giving food in the same plate; pecking on feces of sick poultry; from saliva, feces, blood, offal, skin, feathers of sick or poultry died of disease]*	- Migratory bird	2^b^	(5)	- If kept in same place with sick poultry - Eating from same pot with sick poultry - Scavenging in the feces of sick poultry - Contact with saliva/feces/blood/offal/skin/feathers of sick/dead poultry - Through breathing or air	18^b^	(50)
Can transmit from poultry to humans *[Bird flu can also spread from poultry to human]*		8	(19)		28	(78)
Route of transmission from poultry to humans *[While carrying poultry to the market for selling; while feeding or giving medicine; while slaughtering; while defeathering; if people put their hands in their nose, mouth or eyes without washing after handling sick poultry or poultry that died from disease. The germ of this disease can go inside the windpipe and lungs with breathing through nose]*	- Through consuming bird flu infected poultry/egg - Children’s touching chicken	6^b^	(14)	- Through breathing or air - Through consuming sick poultry - While slaughtering/defeathering sick poultry - Touching own body or children after touching sick poultry - Touching feces of sick poultry	25^b^	(69)
Sign-symptoms in humans *[People can get fever, cold, coughing, throat ache, and breathing difficulty and may even die if the condition worsens]*		0^b^	(0)	- Breathing difficulty - Fever - Coughing - Death - Cold - Damage lungs/kidney/liver	13^b^	(36)

^a^ *N* = Total numbers of interviews with individuals to explore each topic (mentioned in Table 1)

^b^ Frequencies represent number of participants who mentioned at least one of the responses listed in this table

**Table 3:** Awareness of avian influenza prevention before and after intervention, Rajshahi and Chittagong villages, Bangladesh, 2009

Topics [Intervention messages]	Interviews before intervention (*N* ^a^ = 42)			Interviews after intervention (*N* ^a^ = 36)		
	Responses	# of interviews with response	(%)	Responses	# of interviews with response	(%)
Prevention *[Sick poultry should be kept separate and away from healthy poultry or one’s self; sick poultry should not be bought or sold; poultry died from disease should be buried; nose and mouth should be covered well while handling sick and dead poultry and hands should be washed well after handling; sick poultry should not be slaughtered; if one has to slaughter sick poultry, one should: cover nose and mouth very well, stop children from coming near the slaughtering site, cover the blood-smeared slaughtering site with ash or dust and scrape off the soil from that place and bury, wash slaughtering tools well with soap or soda or ash and water; bury offal, skin, feathers, at the end, wash hands with soap or soda or ash and water by rubbing well]*	Any prevention response	8	(19)	Any prevention response	29	(81)
	- Burning/culling	6	(14)	- Covering nose and mouth while slaughtering sick poultry/burying carcass	27	(75)
	- Not consuming sick poultry/egg	4	(10)	- Burying offal, blood, carcasses	27	(75)
	- Cooking/boiling poultry meat/egg well	4	(10)	- Separating sick poultry from healthy poultry and/or humans	25	(69)
	- Handwashing with soap	2	(5)	- Not consuming/slaughtering sick poultry	22	(61)
	- Burying carcass	2	(5)	- Washing hand after slaughtering or handling sick/dead poultry/eggs	22	(61)
	- Not slaughtering/processing poultry	1	(2)	- Keeping children away	13	(36)
	- Using mask	1	(2)	- Cleaning slaughtering site	12	(33)
	- Vaccination	1	(2)	- Not selling sick poultry	9	(25)
	- Not touching carcass with bare hand or using polythene to touch carcass for burying	1	(2)	- Cleaning slaughtering tools	8	(22)
	- Not letting children to touch chicken and wash their hands with soap after touching	1	(2)	- Using polythene/gloves/piece of cloth to touch carcass/slaughter sick poultry	6	(17)
				- Cooking/boiling poultry meat well	6	(17)
				Burning/culling	2	(6)

^a^ *N* = Total numbers of interviews with individuals to explore each topic (mentioned in Table 1)

### Change in preventive practices

Reported preventive practices improved after the intervention for some messages, such as burying the poultry carcass or offal, washing hands with soap, not consuming sick poultry and covering the nose and mouth (Table 4). However, reported practices for other messages, such as separating sick poultry and not selling sick poultry decreased; and cleaning the slaughtering site or tools and keeping children away, remained unchanged. Although participants expressed a willingness to practice the recommended measures immediately after the intervention, the team did not observe changes in behavior during the observation month. Villagers’ reported behaviors were often inconsistent with the observed practices (Appendix 3). The following excerpt from observation notes on disposing a carcass after our intervention illustrates how a raiser justified her actions.

**Table 4:** Reported preventive practices for handling sick poultry by informants before vs after intervention, Rajshahi and Chittagong villages, Bangladesh, 2009

Reported preventive practices	Before intervention		After intervention	
	*n* ^a^/*N* ^b^	(%)	*n* ^a^/*N* ^b^	(%)
Separated sick poultry from healthy poultry/humans	22/27	(81)	25/33	(76)
Did not sell/buy sick poultry	10/20	(50)	9/22	(41)
Buried carcasses or offal/blood of sick poultry	8/46	(17)	16/45	(36)
Did not consume/slaughter sick poultry	2/42	(5)	7/49	(14)
Cleaned site/tools after slaughtering sick poultry	5/8	(62)	8/13	(62)
Washed hand with soap after slaughtering/handling sick/dead poultry	3/9	(33)	6/16	(38)
Covered nose/mouth while slaughtering/handling sick/dead poultry	0	(0)	2/14	(14)
Kept children away	0	(0)	0	(0)

^a^ *n* = Number of interviews where villagers reported practicing the specific method as a preventive measure

^b^ *N* = Total numbers of interviews with individuals to explore each topic (mentioned in Table 1)

5.30 pm: The woman came to our house and informed (the research team) that a chicken with a tumor in its throat died. I (the observer) went with her to her house and saw that she kept the carcass covered with a bamboo basket in the yard… She informed me that she would dump it in the open field at night after completing her household chores. She indicated that if a wildcat took it away it would not stink. She would not dig a pit to dispose of the chicken because it would take a lot of labor. Her husband was an aged person and her son was not at home. Her daughter refused doing the laborious digging. 8.25 pm: She tore a piece of banana leaf and held the carcass with the leaf because it was repulsive to her and to avoid getting a bad smell on her hand. She took the carcass away from her house and dumped it in an open field for the wildcats. She washed her hand with soap and water after going home because the carcass’s wings had come into contact with her hands, which repulsed her.

During the poultry die-off of an unknown cause in the Rajshahi village in 2010, villagers reported that 59 poultry became sick. Of the 59 sick poultry, 21 (36 %) died, 23 (39 %) were slaughtered, eight (14 %) were sold and seven (12 %) were kept under observation with the expectation that they would recover. Although the villagers reported drowsiness and swollen wattles in their poultry among other signs, they did not relate this die-off with avian influenza because they did not see any change in the color of the poultry’s comb or shank. They considered this a regular die-off typical of those that occurred in winter. The team did not observe villagers practicing the recommended behaviors and the villagers did not report the die-off to the livestock office.

### Rationale for ignoring recommended behaviors

#### Perception of risk and financial concern

Villagers commonly reported that the perceived absence of bird flu in their flocks was a reason for not following the recommendations. When a boy, who slaughtered a duck, was asked why he did not cover his nose and mouth while slaughtering, he replied that people should cover their nose and mouth while slaughtering sick poultry and his duck was not sick. While disposing of a dead chicken, a woman said,

“They (the intervention team) told us to bury our poultry only if they get bird flu. But this chicken had a tumor.”

During dissemination, the villagers shared that they had been consuming sick poultry for a long time and had not gotten sick. The intervention team explained the risk of slaughtering, defeathering and processing sick poultry before cooking. However, after the dissemination villagers continued to state that cooking would remove anything harmful from the poultry. This was reflected in their statements below.

“Why shouldn’t we consume sick poultry, when we have raised them for such a long time? We will consume (poultry). Poison becomes water in fire.”

“They (intervention team) told us to dump the sick poultry. Nobody dumps. Everybody lies that they won’t eat (the sick poultry). They dump if the sick poultry die inside their poultry shed, otherwise they slaughter them. They won’t let you know.”

Villagers mentioned cost as a reason for not following the recommendations on isolating, not selling and not consuming sick poultry and using soap for handwashing. Raisers shared that they did not have additional space or money to build a separate shed for sick poultry. They explained that not selling or consuming sick poultry would be a financial loss. As a woman said,

“If the poultry is mildly sick or the disease has just started, that poultry can be consumed… We are poor.”

Some villagers mentioned that if they keep sick poultry outside their bedroom, a thief or wild animal might take them away. They also mentioned that they would not know if their poultry were about to die if they left them outside, and consequently, would not be able to slaughter them before dying in keeping with their religion [13]. In Islam, eating animals that have died from natural causes is prohibited [37].

#### Inconvenience and personal discomfort

Inconvenience was another reason mentioned by the participants for ignoring avian influenza prevention messages. Avoidance of time consuming tasks was a common reason for not burying offal or carcass. While discussing the recommendations, a woman said,

“We know all these (recommendations), but we don’t follow them out of laziness. Who will dig the soil? We remain busy at work.”

A raiser said that selling sick poultry was more convenient than following recommendations for safe handling or slaughtering of sick poultry. Some also mentioned that soap was frequently unavailable at washing places.

Personal discomfort was a major reason for not covering the nose and mouth while handling or slaughtering sick poultry. Villagers reported that covering the nose and mouth was a new recommendation which they were not familiar with before the intervention. Informants reported that covering the nose for a long time while working was uncomfortable and troublesome as the cloth periodically became displaced and needed resetting. A woman explained,

“It takes a long time to process a chicken. They (intervention team) recommended covering the nose and mouth throughout the slaughtering, defeathering and cutting and washing meat. Won’t I feel suffocated?”

Covering the nose and mouth before slaughtering sick poultry was also inconvenient because often poultry die quickly leaving no time for such preparation. A woman said,

“Be it a *gamchha* (towel) or *orna* (women’s scarf), sick poultry will die by the time we find it (to cover nose and mouth). Can anyone cover on time? Aklima’s mother slaughtered one (sick poultry) that day. She did not even have time to bring the slaughtering tool from her room.”

#### Social pressure

Villagers reported social pressure for not conforming to recommendations about selling and consuming sick poultry, covering their nose and using soap for handwashing. Some women said that their husbands, mother-in laws or sons rebuked them if they did not slaughter or sell sick poultry before they died. A woman shared that if her poultry died before she could slaughter them, her son would become angry and say,

“Why don’t you slaughter? It would have been a good curry. Why aren’t you more careful?”

The women shared that people might ridicule them if they covered their nose and mouth. As a woman said,

“We never worked while covering our noses and mouths before. If I do it, and you do it and she does it, then it is not a problem. We three can tell five more people. But it cannot be done alone… If a person, who does not know about the recommendation, sees me doing it, that person will certainly laugh at me.”

A woman also shared her concern about being rebuked by her mother-in-law for wasting soap,

“There was a custom of washing hands with soap after touching anything (dirt) in my home. But it is not practiced here (her in-law’s house). If I try to do it, my mother-in-law rebukes me and scornfully says, ‘I brought a soap-user daughter-in-law.”

Nevertheless, villagers also noted that social pressure could be a motivation for following the avian influenza prevention recommendations. During observations and conversations, the team found some villagers, especially children, reminding others about practicing the recommendations. A woman shared that her mother-in-law reminded her of the messages and forbade her to feed sick poultry meat to the children.

While the women of the household were cutting meat, a girl came from the neighboring household and asked, “Will you bury the offal?” Then the woman replied, “Why? Is it a sick duck? We would have had to bury if it were a sick duck.”

A young girl said,

“I asked (my mother) to cover her nose but mother dumped it (carcass) in the pond without covering her nose… A crow took it away.”

#### Skepticism about the necessity of the intervention

Villagers shared that providing treatment to infected poultry would be a more convenient solution for them than following avian influenza prevention practices. When the intervention team recommended that they avoid selling or slaughtering infected poultry or follow safe slaughtering methods, the villagers asked for avian influenza medications for poultry. The intervention team informed the villagers that there was no treatment or medicine available in Bangladesh for avian influenza. The research team found the villagers skeptical about the importance of the recommended preventive practices even after the dissemination. The following quotations summarize their concern.

“Why do you teach the technique of slaughtering (sick poultry) without giving medicine?”

“Talking about these (recommendations) won’t work. Bring medicine.”

“Which doctor should we go to and how should we treat our sick poultry?”

Some informants thought that avian influenza was nothing but a conspiracy or propaganda by foreign countries intended to damage the economy or the poultry industry of Bangladesh. Some of these informants expressed resentment towards the Government of Bangladesh for its role in controlling avian influenza through the culling of flocks.

## Discussion

The villagers’ awareness about avian influenza increased after the intervention, however, the intervention did not result in substantive change in preventive behaviors. Villagers did not consider recommendations, such as isolating, not selling and not consuming sick poultry, burying the offal and carcass, and using soap for handwashing as feasible; while the recommendation to cover their nose and mouth was unacceptable to them. After the intervention, our study participants were aware that the zoonotic transmission of avian influenza was possible through direct contact and through inhalation, which matches with the biomedical perspective about the main transmission pathways of avian influenza infection [38, 39]. Consequently, they were aware that covering their nose and mouth was a preventive measure, which also agrees with the biomedical perspective.

By attempting to describe and interpret the behaviors and beliefs of the communities captured through diverse ethnographic tools, this study provides an understanding of why villagers’ practices did not change after the intervention despite increased awareness. First, financial loss was an important concern for poultry raisers. They sold or consumed sick poultry because these were a source of food and cash in-hand particularly for women [14, 29]. Our observations are consistent with Leppin and Aro (2009) who found that people living under economically precarious conditions may place a relatively low value on health consequences for speculative and distant threats compared to more immediate daily economic hazards [40].

A second important factor for not adopting avian influenza prevention practices was low perception of risk. Absence of immediate negative consequences, i.e., evidence of transmission of avian influenza or other poultry disease to humans, might cause unaffected communities to have a lower estimate of the risk of avian influenza than communities with outbreaks [40]. Bangladeshi villagers discount the risk of zoonotic transmission of avian influenza [14]. To a community without an outbreak, the risk of a disease like avian influenza was perceived as low. These determinants are important preconditions for backyard raisers’ perception of risk and could affect an individual’s willingness to engage in behaviors intended to reduce their risk [41]. Until villagers believe that avian influenza could affect them, they will not be willing to change their practical approaches that they have been practicing for many years [29]. Compared to avian influenza, small commercial poultry farmers were more concerned about Newcastle disease and infectious bursal disease, which caused mortality in their flock [42]. In Bangladesh, agents contributing to death among chickens are Colibacillosis (28 %), Salmonellosis (14 %) and Newcastle disease (11 %) [43]. However, such infections have little or no impact on human health.

Villagers perceived that cooking eliminated infectiousness, which might have been further strengthened by the government’s recommendation that cooked eggs and meat are safe to consume [10]. This perception undermines the risk of transmission that can occur during the preparation phase. Moreover, villagers did not believe that avian influenza occurred in their flock until they could identify signs of the disease particularly through changes in the color of comb and shank. The problem with identifying avian influenza only through observation, however, is that most signs of avian influenza are similar to those of other commonly occurring poultry diseases, such as Newcastle disease [44]. Therefore, future preventive recommendations could be broadened to include other diseases, such as Newcastle diseases, that the farmers are concerned about, rather than focus only on avian influenza.

A third reason for not adopting prevention recommendations was inconvenience and physical discomfort. Villagers found burying sick poultry carcasses or offal a too laborious recommendation to comply with and preferred either keeping sick poultry close to them or selling them off instead of following safe slaughtering methods. Villagers repeatedly noted that covering their nose and mouth caused the sensation of suffocation. Other studies have reported inconvenience as an important barrier for adoption of other public health interventions, including using insecticide-treated mosquito nets or improved cookstoves [45, 46]. One of the reported reasons bed nets for malaria prevention failed in one area in Mozambique was because people felt hot under the net and chose to sleep outside the net’s cover [47].

Social pressure or the fear of being rebuked or ridiculed by others in the family or community, also worked as a barrier to adopting certain recommended behaviors. Changing behavior does not involve an individual in isolation but rather is influenced by family, neighborhood or a community collectively. Jenkins and Curtis reported avoidance of shame and embarrassment as an important driver for installing a latrine in rural Benin [48]. A review study that assessed the role of factors influencing adoption of insecticide-treated mosquito nets, household water treatment and improved cookstoves reported that health promotion motives alone are insufficient to drive adoption and sustainable use of prevention measures; non-health factors such as convenience, comfort and sociocultural factors dominate adoption of behavior [49]. Thus, social pressure and norms could be utilized to motivate villagers to adopt new behaviors aimed at preventing avian influenza transmission.

Lastly, villagers perceived treatment and medicine as a more convenient solution rather than complex behavior change. However, there is no treatment available for avian influenza infection in poultry. Although not a treatment, some countries have used avian influenza vaccination as an intervention to reduce the risk of transmission. Some countries have used these vaccines on a long-term basis, some have used it temporarily, while others have not used it at all [50]. The Drug Administration authority of the Government of Bangladesh has allowed three pharmaceuticals to import avian influenza vaccines [51]. At the time of this writing, the Government of Bangladesh is in the process of developing an H5N1 vaccination policy for commercial poultry [50]; other livestock vaccines have had low uptake in the backyard sector in Bangladesh [52, 53] and so we would not expect widespread uptake of an H5N1 vaccine among backyard poultry producers.

A systematic review of biosecurity measures for backyard poultry in developing countries highlighted the importance of biosecurity for backyard poultry to reduce the spread of HPAI, and reported a lack of biosecurity guidelines, lack of research on the impact of biosecurity measures in backyard settings and a lack of evidence of their feasibility and effectiveness [54]. Educational interventions to improve biosecurity have typically been used to change behaviors among backyard poultry raisers [2–7, 55]. A study in Laos assessed the impact of countrywide intensive educational campaigns on awareness and behavior related to HPAI in 2007 and reported higher rates of cessation of poultry consumption and dead poultry burial but no improvement in poultry raising practices, immunization or reporting mortality to authorities compared to 2006 [7]. The authors discussed the risk of misinterpretation and misconception, since the content and accuracy of the messages reported by the participants differed widely depending on training exposure.

A community-based education trial to improve backyard poultry biosecurity in rural Cambodia highlighted the success of a cascade training approach by involving community members to raise awareness of avian influenza [3]. However, the authors discussed difficulties relating to feasibility of major scale-up of the intervention and maintaining accuracy of the messages disseminated through this method. The study also showed that despite improvement in knowledge and reported practices relating to biosecurity, appropriate practices commonly remained infrequent. Educational interventions to promote standard behaviors in Cambodia and Vietnam also found minimal or no positive change in behavior [2, 6]. In Bangladesh, the government disseminated messages through mass media and workshops or training at livestock offices. These interventions, which were more passive than our interpersonal intervention, either did not evaluate the impact through observations or did not explore reasons for their lack of impact. Our study attempted to modify recommendations to make them more context-appropriate and answer why such educational interventions are unlikely to change behavior, even after increasing awareness.

Our intervention had limitations. Although the intervention team referred to both sick ducks and sick chickens as ‘sick poultry’, our intervention was not designed to include discussion on asymptomatic ducks, which may be reservoirs of avian influenza [56], and therefore, did not account for risks of HPAI from all poultry. To reduce the risk that the recommended practices would be overwhelming, we sought to change a specific high-risk activity, i.e., slaughtering of sick poultry. Another limitation of the intervention was the short length of time allocated for dissemination and that no reinforcement was provided to promote meaningful behavior change. Nevertheless, this study provides an in-depth understanding of the underlying reasons rural raisers do not comply with such recommendations.

Despite our effort to make the intervention team appear unrelated with the research team, the villagers often correctly assumed that they worked together. This might have caused some courtesy bias, which may have been reflected in some informants’ reporting adopting the preventive methods. However, observed practices did not change after the intervention, so we believe courtesy bias did not undermine our primary conclusion that the intervention did not foster the adoption of recommended avian influenza prevention practices.

We conducted this qualitative study in only two small communities which were unlikely to represent the entire country. Nevertheless, these communities have common socio-economic features with other rural backyard poultry raising areas of Bangladesh [14, 29]. Study populations were not enrolled to be strictly representative of a broader population, so quantitative comparisons should be interpreted with caution.

## Conclusions

This study suggests that despite an intense interpersonal intervention which raised awareness about avian influenza and recommended prevention practices, there was little or no change in behavior among backyard poultry raisers. Poultry raisers chose to ignore the recommendations because they were perceived as impractical and unacceptable. Interventions that focus only on health benefits often ignore financial cost, convenience and comfort and consequently are unlikely to succeed. Incorporating non-health benefits to such interventions may increase their adoption. Approaches that minimize personal discomfort and interrupt the transmission route or are marketed as a means of improving return on investment through the raising of healthy poultry should be developed and piloted for efficacy and acceptability in endemic countries. Messages on hand washing and cleaning of slaughtering tools and the slaughtering site could be promoted as routine practices and included in the government’s campaign for general food hygiene and safety.

## Appendix 1. Guideline for observation

A. Practices related to sick and dead poultry:
  - Observe the activities regarding sick poultry handling (at the time of feeding, giving medicine, transporting for selling or separating from other poultry).
  - Observe what happens to the excreta of the sick poultry.
  - Observe sick poultry slaughtering, defeathering, cutting into meat, preparation for consumption (including personal hygiene and cleaning of instruments and the area, where slaughtering and processing of poultry take place, after slaughtering, processing, preparation, handling and dumping of poultry offal) and record all the activities in detail, especially those which involve physical contact.
  - Observe the practice relating to dead poultry (hint: bury under the soil, through in the water or bush).
  - Observe children’s involvement in sick poultry handling and slaughtering (such as playing with or giving medicine to the sick poultry, playing with the raw poultry meat while processing after slaughtering, assisting in slaughtering or processing or cleaning)
  - Observe who participate most of the time in sick poultry slaughtering and handling of sick and dead poultry (male or female or children).
  - Record hand washing practice after sick poultry slaughtering and handling of sick and dead poultry.
  - Observe their interaction or contact with others or the environment after slaughtering sick poultry or handling sick or dead poultry.
B. Information related to the intervention:
  - Observe if they follow the messages while sick poultry slaughtering and handling of sick and/or dead poultry or not and up to what extent they follow.
  - To conform to the messages, observe whether they use any alternative technique that is convenient for them to follow.

## Appendix 2. Guideline for informal interview

A. Awareness about avian influenza:
  - Explore what they know about ‘a disease in which many poultry died in Bangladesh during 2007-08’ or ‘bird flu’ (probe sign-symptoms, routes if transmission from poultry to poultry and poultry to humans, preventive measures, and source of information).
B. Practices related to sick and dead poultry:
  - Explore the caring practice related to sick poultry.
  - Explore the treatment seeking pattern for poultry sickness (probe nature and place of treatment, source of information about disease).
  - Explore detail about sick poultry slaughtering, defeathering, cutting into meat, preparation for consumption (probe personal hygiene and cleaning of instruments and the area where slaughtering and processing of poultry take place, after slaughtering, processing, preparation, handling of organs and entrails and dumping of waste).
  - Explore the practice regarding dead poultry (hint: bury under the soil, through in the water or bush, etc.).
  - Explore their explanation behind each relevant activity while sick poultry slaughtering and/or handling sick and/or dead poultry.
C. Information related to the intervention:
  - Explore if they can recall the messages.
  - Explore if they can relate any change in their behavior for the messages.
  - Explore their perception regarding the messages and their response to those. Explore whether the messages were convenient, feasible and culturally appropriate for them (discuss each message separately).
  - Explore their opinion regarding the approach of message dissemination.
  - Explore what difficulties/problems they face to follow the messages and what changes they suggest in the messages to overcome those (discuss each message separately and ask what and how they would prefer those to be told. Explore what they share spontaneously, without being asked directly).

## Appendix 3

**Table 5:** Observed preventive practices for handling sick poultry before vs. after intervention, Rajshahi and Chittagong villages, Bangladesh, 2009

Observed practices	Before intervention *n*/*N* ^a^	After intervention *n*/*N* ^a^
Sick poultry were kept:		
Separate from healthy poultry and humans (recommended)	0/3	0/2
Separate from healthy poultry	2/3	1/2
Together with healthy poultry/humans	1/3	1/2
Sold/bought sick poultry	0/0	2/2
Carcasses of sick poultry were:		
Buried under soil (recommended)	0/3	0/3
Thrown in open place/water body	33	3/3
Consumed/slaughtered sick poultry	3/6	4/8
Sick poultry slaughtering site was:		
Covered blood with ash/dust and then scraped off and buried the soil (recommended)	0/3	0/4
Poured water on blood	2/3	2/4
Not cleaned	1/3	2/4
Sick poultry slaughtering tools were:		
Washed with soap or soda or ash (recommended)	0/3	0/4
Rinsed with water	2/3	1/4
Not cleaned	1/3	3/4
Offal/blood of sick poultry was:		
Buried under soil (recommended)	1/3	0/4
Thrown in open place/water body	2/3	4/4
Hands after slaughtering/handling sick/dead poultry were:		
Washed with soap (recommended)	1/6	1/9
Rinsed with water	5/6	6/9
No handwashing	0/6	2/9
Nose/mouth while slaughtering/handling sick/dead poultry were:		
Covered with a piece of cloth (recommended)	0/6	0/9
Not covered	6/6	9/9
Children were:		
Kept away from slaughtering site (recommended)	0/3	0/4
Present in slaughtering site	3/3	4/4

^a^ *n* = Number of sessions where villagers were observed to perform practices mentioned in the first column

*N* = Total number of opportunities to observe the practices pertinent to each topic (mentioned in Table 1)
